# In vitro maturation of ovine oocyte in a modified granulosa cells co-culture system and alpha-tocopherol supplementation: effects on nuclear maturation and cleavage

**DOI:** 10.1186/s40781-015-0061-5

**Published:** 2015-08-14

**Authors:** Hamideh Adeldust, Saeed Zeinoaldini, Hamid Kohram, Mahmoud Amiri Roudbar, Morteza Daliri Joupari

**Affiliations:** Department of Animal Science, College of Agriculture and Natural Resources, University of Tehran, Karaj, Iran; Department of Clinical Sciences, Faculty of Veterinary Medicine, Shahid Chamran University, Ahvaz, Iran; Department of Animal Science, Shahid Bahonar University of Kerman, Kerman, Iran; National Institute of Genetic Engineering and Biotechnology, Tehran, Iran

**Keywords:** Granulosa cell, In vitro maturation, In vitro fertilization, Embryo

## Abstract

This study was designed to investigate the effects of α-tocopherol and granulosa cells monolayer on nuclear maturation and cleavage rates of ovine cumulus-oocyte complexes (COCs). The COCs (*n* = 2814) were matured in maturation medium supplemented with various concentration of α-tocopherol (0, 5, 10, 15 μg/ml), oocytes were incubated at 39 °C with 5 % CO_2_ for 24 h in three culture systems: (a) maturation medium (MM; *n* = 884), (b) co-cultured with granulosa cells (CG; *n* = 982) and (c) co-cultured with granulosa cells and cells were further cultured in MM for 12 h (CG + 12hMM; *n* = 948). Our results showed that α-tocopherol had no effect on GVBD and MII as compared to control group, but when α-tocopherol added to maturation medium the rate of cleavage decreased. This indicates interaction of above mentioned factors in any of the treatments showed no significant differences on the rate of maturation and cleavage stages (MII, GVBD and cleavage) (*p* > 0.05). The oocytes co-cultured with granulosa cells for 24 h had beneficial effects on cleavage rate. The maximum MII and cleavage rates were achieved when oocytes had extra 12 h culture in the maturation medium without granulosa cells. Results also showed our modified co-culture system (CG + 12hMM), improved rates of MII and the cleavage in comparison with other studied maturation systems.

## Introduction

Early stages of embryo development depend on maternally syntheses of proteins and mRNAs which were produced during oocytes growth and maturation [1]. It is accepted that production of in vivo embryos has higher quality than in vitro embryos [2]. Nuclear and cytoplasmic maturation of the in vivo oocytes are generally dependent on follicular fluid, interaction between follicular cells, oocytes and endocrine systems [3]. The oocytes in preantral follicles are not capable to resume meiosis. At this stage, oocyte-granulosa cell gap junctions may provide biosynthetic substrate to oocyte to complete their growth [4, 5]. However, in vitro embryo production, the COC is separated from the maternal follicle and transported to the maturation medium. Usually, the goal of maturation medium is to simulate the ovarian follicular environment. To date many efforts have been made to improve the oocyte maturation by using COC co-culturing with granulosa cells [6]. Applying co-culture systems (with different types of somatic cells) during IVM could be useful for oocyte maturation and embryo development in cattle [7, 8]. Further research showed that granulosa cells in rabbit oocyte maturation medium caused 3 h deceleration of nuclear maturation, which probably allowed a better coordination between nuclear and cytoplasmic maturation, improving subsequent embryonic developmental stages. Indeed, increasing the quality of IVM medium may improve the in vitro culture systems of embryos [9, 10].

Reactive oxygen species (ROS) are harmful for embryo development and probably makes numerous types of embryo damages, including embryo cell block and apoptosis [11]. α-tocopherol is a predominant lipid-soluble antioxidant which can have a powerful function as an antioxidant in vitro to protect the cells from ROS [12]. It was reported that generating the oxygen radicals by addition of 2,2′-azobis(2-amidinopropane) dihydrochloride (AAPH) in culture medium caused lower rate in blastocyst development [13]. Moreover, supplementation of the culture medium by α-tocopherol can improve geranulosa cells performance and viability [14]. Accordingly, it is possible that improving geranulosa cells operation by α-tocopherol affects the co-culture system and improves preimplantaion development. This study was designed to investigate: (i) the effect of α-tocopherol in maturation medium, (ii) the effect of co-culture of COCs with granulosa cells monolayer, and (iii) the interaction between α-tocopherol and granulosa on maturation and cleavage rates of ovine oocytes.

## Methods

All chemicals and reagents were purchased from Sigma Aldrich Company (Germany), unless otherwise specified.

### COCs collection

Ovine ovaries (*n* = 1362) were collected from animals slaughtered at commercial slaughterhouse and transported within 1–2 h to the laboratory in saline solution (0.9 % NaCl) supplemented with 50 μg/ml gentamycin sulfates at 30–35 °C. Ovaries were washed in normal saline solution and extraneous tissues were removed. COCs were gently aspirated from 2–6 mm follicles using 10 ml syringe with 20-gauge needle. The oocytes with homogenous cytoplasm were selected and washed three times in aspiration medium (TCM-199-Invitrogen) supplemented with 3 mg/ml BSA, 20 mM sodium pyruvate and 50 μg/ml gentamycin sulfates).

### In vitro maturation (IVM)

The COCs were washed three times in maturation medium (TCM199 with culture media containing 10 % FBS, 20 mM sodium pyruvate, 0.5 mg/ml FSH, 5 mg/ml LH, and 50 μg/ml gentamycin sulfates and 1 μg/ml estradiol). The COCs (*n* = 2814) were randomly placed in maturation medium supplemented with various concentration of α-tocopherol. In the first group (MM), 20 COCs were placed in 100 μl droplets of maturation medium supplemented with 0, 5, 10, 15 μg/ml α-tocopherol and incubated for 24 h. In the second group (CG), 18–20 COCs were placed in four well petri dish containing 0.5 ml maturation medium on a monolayer of granulosa cells (cells were previously cultured for 7 days to achieve monolayer) with different concentration of 0, 5, 10, 15 μg/ml α-tocopherol and incubated for 24 h. In the third group (CG + 12hMM), oocytes were cultured on a monolayer of granulosa cells for 24 h and transported to MM for additional 12 h incubation. Droplets are covered with mineral oil and incubated at 39 °C with 5 % CO_2._ After maturation period, COCs (*n* = 1512) were stained with Hoechst 33342 to determine the rate of germinal vesicle break down (GVBD) and MII stages in different maturation media [15]. Remaining COCs (*n* = 1302) were used for IVF and IVC procedures.

### In vitro fertilization (IVF)

Following IVM, the COCs were washed three times in TCM199 supplemented with 3 mg/ml BSA and placed into 50 μl droplets of fertilization medium (IVF-TALP supplemented with 25 μg/ml heparin and 6 mg/ml fatty acid free BSA). Ovine testes were collected from slaughtered animals at commercial slaughterhouse in a normal saline solution supplemented with 50 μg/ml gentamycin sulfates and transported to the lab within 1–2 h at 30–35 °C. After semen extraction, epididymal motile sperm cells were collected and washed two times in HEPES TALP medium and separated by swim up method. In each fertilization drop a concentration of 1 × 10^6^/ml spermatozoa were added.

### In vitro culture (IVC)

Following IVF, cumulus cells were separated from presumptive zygotes by gently pipetting. Denuded zygotes were washed twice in culture medium (SOF supplemented with 10 ng/ml EGF, 50 μg/ml gentamycin sulfates and 5 % FBS). To determining the rate of cleavage, 8–10 zygotes were cultured in each four-well petri dish containing 250 μl of culture medium under mineral oil at 39 °C with 5 % CO_2_.

### Statistical analyses

Data were analyzed as a factorial experiment based on a completely randomized design with 2 factors and triplicates. Analysis of variance was used to evaluate the main effects of α-tocopherol (4 levels) and maturation methods (3 systems) and their interactions (Table 1). Duncan’s Multiple Range Test with significance defined at *p* < 0.05, was used to determine differences among means. Analysis of variance was conducted using GLM procedure of SAS software version 9.1 (SAS, 2004).

**Table 1:** Data of the factorial experiment with four and three levels of α -tocopherol and the maturation culture system, respectively

Maturation medium	α**-**tocopherol (μg/ml)	No. of oocytes (maturation/cleavage)	GVBD (% ± SEM)	MII (% ± SEM)	Cleavage (% ± SEM)
MM	0	122/88	18 (14.75 ± 3.40)	80 (65.57 ± 5.84)	32 (36.36 ± 7.37)
	5	115/120	15 (13.04 ± 2.49)	81 (70.43 ± 4.28)	43 (35.83 ± 4.77)
	10	101/114	16 (15.84 ± 4.30)	64 (63.37 ± 5.05)	47 (41.23 ± 6.05)
	15	118/106	27 (22.88 ± 3.43)	67 (56.78 ± 2.47)	32 (30.19 ± 1.57)
CG	0	135/116	58 (42.96 ± 4.89)	36 (26.67 ± 3.78)	67 (57.76 ± 3.00)
	5	125/140	47 (37.60 ± 3.46)	51 (40.80 ± 4.20)	76 (54.29 ± 2.26)
	10	135/119	54 (40.00 ± 3.07)	52 (38.52 ± 1.21)	55 (46.22 ± 3.16)
	15	135/77	48 (35.55 ± 1.12)	49 (36.30 ± 4.80)	26 (33.77 ± 2.27)
CG + 12hMM	0	131/130	11 (8.37 ± 3.45)	112 (85.50 ± 3.37)	79 (60.77 ± 4.08)
	5	131/91	6 (4.58 ± 1.88)	115 (87.79 ± 4.17)	47 (51.65 ± 1.53)
	10	138/82	9 (6.51 ± 1.91)	117 (84.78 ± 4.57)	57 (69.51 ± 3.47)
	15	126/119	11 (8.73 ± 2.73)	105 (83.33 ± 1.30)	57 (47.90 ± 1.03)

## Results

### Effects of α-tocopherol and different types of IVM media on oocytes maturation

**Fig. 1:**
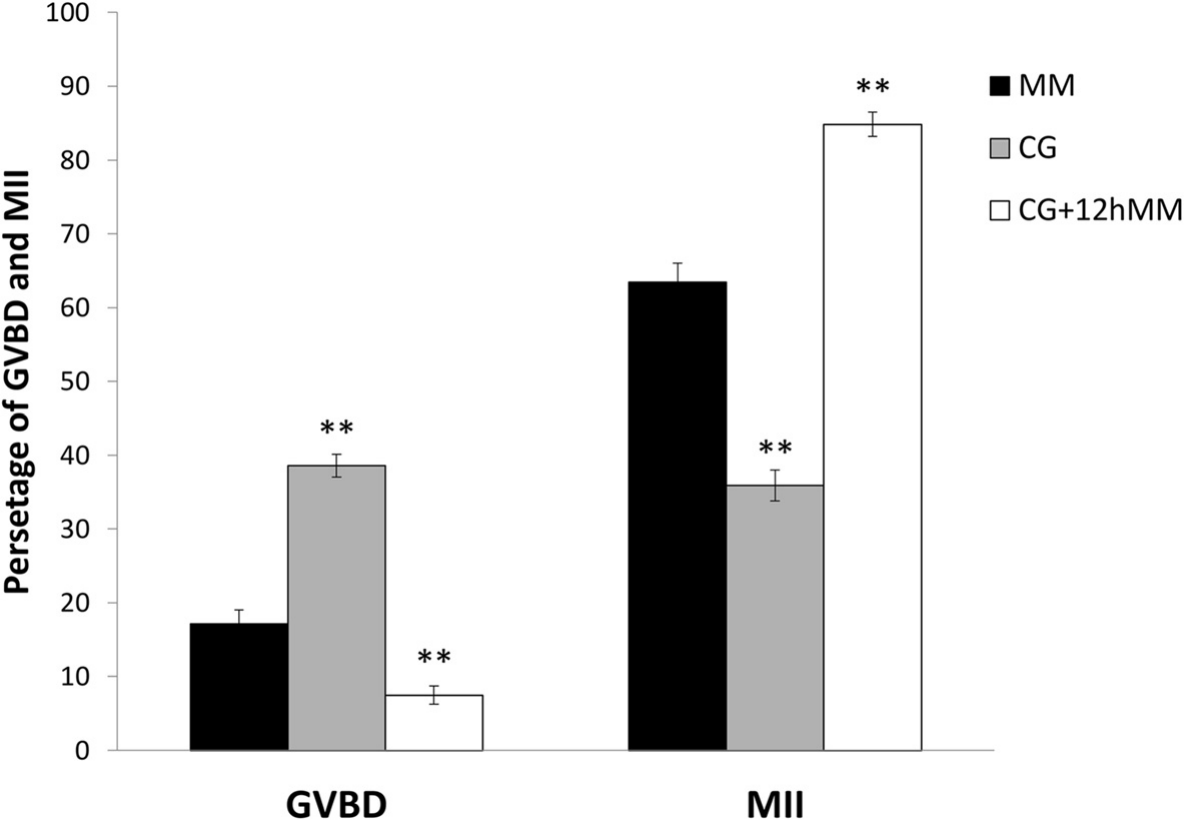
The rates of GVBD and MII stages in different culture systems. The different culture systems include maturation medium (MM) as control, co-culture with granulosa cells for 24 h (CG) and co-culture with granulosa cells plus 12 h further culture in MM (CG + 12hMM). ** Statistically different from the respective controls, *p* < 0.01. Bars represent mean ± SEM. Numbers of oocytes in each treatment: MM (*n* = 456), CG (*n* = 530), CG + 12hMM (*n* = 526)

Interaction between the two main factors (α-tocopherol and type of IVM medium) in any of the treatments showed no significant difference on rate of oocyte maturation as far as GVBD and MII stages are concerned (*p* > 0.05). The concentrations of α-tocopherol used in IVM medium had no effect on GVBD and MII rates of oocytes as compared to control group (Table 2). Oocytes co-cultured with granulosa cells monolayer (CG), showed a decrease in MII rates (Fig. 1) as compared to the maturation medium system (MM) (*p* < 0.01). Nevertheless, transferring of the oocytes to the maturation medium and further 12 h incubation significantly increased the numbers of oocyte that reached to MII stage (*p* < 0.01) (Fig. 1). Conversely, the rate of GVBD in CG and CG + 12hMM increased and decreased respectively (*p* < 0.01) as compared to control.

**Table 2:** The main effects of various concentrations of α-tocopherol supplementation on GVBD and MII stages

α-tocopherol (μg/ml)	No. of oocytes	No. of GVBD (%)	No. of MII (%)
0	388	83 (21.39)	230 (59.27)
5	371	69 (18.59) ^ns^	246 (66.30) ^ns^
10	374	80 (21.39) ^ns^	230 (61.49) ^ns^
15	379	86 (22.69) ^ns^	221 (58.31) ^ns^
±SEM		1.83	2.32

Ns in the same columns denotes a non-significant difference (*p* > 0.05) as compared to control (0 μg/ml)

### Effects of α-tocopherol and different IVM systems on the cleavage rate

No interactional effect between the two main factors on cleavage rate was observed, but there was a decrease on cleavage rate (*p* < 0.05) in oocytes matured in 5 and 15 μg/ml α -tocopherol supplemented medium as compared to control (Table 3).

**Table 3:** The main effects of various concentrations of α-tocopherol supplementation on cleavage stage

α-tocopherol (μg/ml)	No. of oocytes	No. of cleavage (%)
0	334	178 (54.29)
5	351	166 (46.36)^a^
10	315	159 (50.37)^ns^
15	302	115 (37.95)^a^
±SEM		3.74

Ns and^a^ in the same columns denote not significant (*p* > 0.05) and significant (*p* < 0.05) differences, respectively, as compared to control (0 μg/ml)

**Fig. 2:**
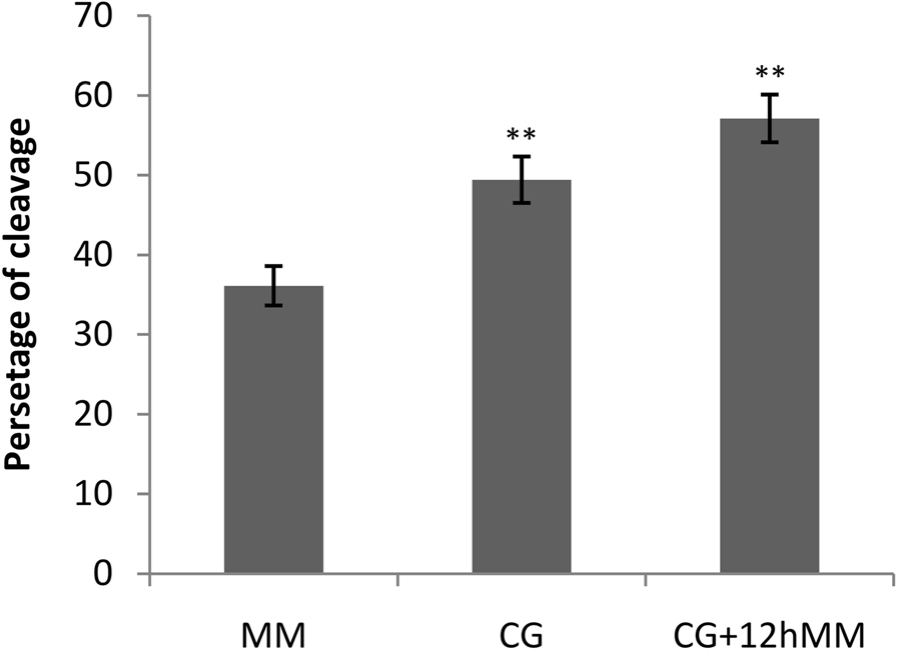
The rates of cleavage in different culture systems. The different culture systems include maturation medium (MM) as a control, co-culture with granulosa cells for 24 h (CG) and co-culture with granulosa cells plus 12 h further culture in MM (CG + 12hMM).). ** Statistically different from the control, *p* < 0.01. Bars represent mean ± SEM and distinct letters on bars are significantly different (*p* < 0.01). Numbers of oocytes in each treatment: MM (*n* = 428), CG (*n* = 452), CG + 12hMM (*n* = 422)

The cleavage rate was higher (*p* < 0.01) in oocytes matured in both maturation media (CG + 12hMM and CG) containing granulosa monolayer cells than maturation medium (MM) without granulosa cells. An extra 12 h culture of COCs in the maturation medium increased the cleavage rate (*p* < 0.01) in comparison with the oocytes cultured just on granulosa monolayer cells for 24 h (Fig. 2).

## Discussion

In bovine, additional α-tocopherol in maturation medium had no influence on nuclear maturation, fertilization and blastocyst production rates [16, 17]. In buffalo, maturation medium supplemented with vitamin E, in 5 % O_2_ showed no effect on embryo development, whereas embryos cultured under 20 % O_2_ increased the rate of blastocysts [18]. In ovine, supplementation of vitamin E in maturation medium had no effects on oocyte maturation and embryonic preimplantation development [19]. Nevertheless, some data showed that vitamin E supplementation in the culture medium increased the rate of expanded blastocysts [20]. The result of the present study revealed that supplementation of maturation medium with α-tocopherol had no effect on oocyte maturation rate when incubated in 5 % O_2_ at 39 °C for 24 h. A part from oocyte maturation, supplementation of maturation medium with 10 μg/ml α-tocopherol also had no effect on cleavage rate. However, maturation medium with a high dose of α-tocopherol showed a deleterious effect on cleavage rates. Although an improvement of the geranulosa cells performance had been observed in the supplemented medium with α-tocopherol [14], there was no interaction between α-tocopherol and geranulosa on the rates of maturation and cleavage of ovine oocyte.

Co-culture of the granulosa cells with COCs, may produce series of substances in vitro, that inhibit meiosis and delay the meiotic maturation. It is documented that midkine is secreted from granulosa cells under control of gonadotropins [21]. This factor enhances the ability of oocytes to reach the blastocyst stage, but has no effect on meiotic maturation [22]. In bovine, granulosa cells have inhibitory effect on oocyte maturation [23]. The homologous gap junctions between the oocytes and cumulus cells cause transfer of cAMP from one cell to the other cells. Consequently, cAMP accumulation in the oocytes, stimulates protein kinase enzymes, which in turn inhibit the meiosis [24–26]. Nevertheless, these cell communications may coordinate the nuclear and cytoplasmic maturation of the oocytes [4]. In this study, co-culture of the oocytes with granulosa cells indicated a delay in MII phase. This delay made an increase of the GVBD’s rate in MM medium with considerable decrease after further culture in maturation medium for 12 h (MM + 12hMM). In other words, more than half of the oocytes arrested in GVBD stage after 24 h co-culture with granulosa, but extra culturing of the oocytes in MM permits some of them to complete their meiosis. The dramatic increase in the GVBD’s rate in the co-culture system (CG), which is probably accurse due to cell contact between oocyte and granulosa [7], may allow a better coordination between nuclear and cytoplasmic maturation in which improve maturation potential. Consequently, this coordination makes an increase in percentages of the matured oocytes that cultured further in maturation medium for 12 h when it was compared to culturing of oocytes in maturation medium with or without granulosa cells for 24 h only.

Presence of granulosa cells in maturation medium improved maturation, fertilization and embryo development in sheep [6]. In caprine, monolayer of granulosa cell caused higher rate of maturation compared to co-culture with mass-granulosa cells [27]. Whereas, co-culture of the oocytes with granulosa cells during maturation period, had no effect on the rate of mice oocyte maturation [28]. Supplementation of small follicular wall in maturation medium also increased bovine oocyte capacity to reach the morula stage [29]. So, using granulosa cells for in vitro maturation can delay the meiotic maturation. Hence, oocytes may obtain more capabilities during IVM and in turn, better results will be gained at subsequent preimplantaion stages [30]. In this study, granulosa cells supplementation in maturation medium increased the cleavage rate, with or without extra culture in maturation medium for 12 h. Furthermore, higher percentage of cleavage was achieved when oocytes were cultured further in maturation medium for 12 h after 24 h culturing on monolayer granulosa cells (MM + 12hMM).

## Conclusions

In conclusion, supplementation of α-tocopherol in maturation medium had no beneficial effect on MII and cleavage rate. Presumably, there could be no interaction between α-tocopherol and co-culture system on the maturation and cleavage rate. Moreover, culturing on a monolayer of granulosa cells in maturation medium for 24 h increased the cleavage rate of ovine oocytes. Here, we designed a new co-culture system (CG + 12hMM) in which MII and the cleavage rates were highly improved in comparison with other maturation systems.
